# Supplementary material for: Patient-reported physical functioning is limited in almost half of critical illness survivors 1-year after ICU-admission: A retrospective single-centre study
Source: PLoS One. 2020 Dec 14;15(12):e0243981. doi: 10.1371/journal.pone.0243981 (PMC7735575; doi:10.1371/journal.pone.0243981)
Supplement: S1 Table — (DOCX) [file pone.0243981.s001.docx]

**S1 Table. Percentage of missing values and medians of the original dataset and the pooled imputed datasets**

| **Variable** | **Missing values, n (%)** | **Median original dataset** | **Median imputed dataset** |
| --- | --- | --- | --- |
| 3 Months |  |  |  |
| MMI | 104 (41.6) | 85 [74-85} | 85 [74-88] |
| Hand grip strength | 54 (21.6) | 96.15 [81.15-116.80] | 96.00 [80.68-116.96] |
| BIS | 31 (12.4) | 20 [20-20] | 20 [20-20] |
| 6-MWT | 76 (30.4) | 86.00 [68.75-97.00] | 86.00 [67.99-97.00] |
| BBS | 74 (29.6) | 53 [49-56] | 53 [49-56] |
